# Retention and efficacy of long-lasting insecticide-treated nets distributed in eastern Sudan: a two-step community-based study

**DOI:** 10.1186/1475-2875-7-85

**Published:** 2008-05-20

**Authors:** Saad El-Din Hussein Hassan, Elfatih M Malik, Somia I Okoued, Elsadig M Eltayeb

**Affiliations:** 1 Emergency and Humanitarian Action Directorate, Federal Ministry of Health, Khartoum, Sudan; 2 Communicable Disease Control Directorate, Federal Ministry of Health, Khartoum, Sudan; 3 Communicable Disease Control Directorate, State Ministry of Health, Kassala, Sudan; 4 Expanded Programme of Immunization, Federal Ministry of Health, Khartoum, Sudan

## Abstract

**Background:**

In order to assess the effectiveness of long-lasting insecticide-treated nets (LLINs) as a method for malaria control, there is a need to determine how high is the retention of bed nets, how they are utilized, and how efficacious they are against the mosquitoes that transmit the disease. This is especially important in case of Sudan after emergence of resistance to pyrethroids in use.

**Methods:**

This two-step study aimed to assess the retention and efficacy of LLINs (Olyset™) distributed in the year 2006 in Kassala district in eastern Sudan. In the first step, using a cluster sample technique, heads of 210 households (30 by 7) were interviewed, and six LLINs were collected and later tested for efficacy. In the second step, eight focus group discussion sessions were conducted to complement the results from the first step.

**Results:**

Results showed that the retention of LLINs was 92.9% one-and-half years after distribution. Some bed nets were distributed against a price. Utilization of bed nets by children under five years of age and by pregnant women was found to be 55% and 42.1% respectively. For the bioassay efficacy tests, mean knock down after 60 minutes was 91.1%, while mortality after 24 hours was 99.4%.

**Conclusion:**

LLINs (Olyset™) were efficacious at the time of the study. People appreciated the usefulness but were not fully aware of their importance and were not motivated enough to use them. The retention of the bed nets was quite high but the utilization of the nets needs more focus from the National Malaria Control Programme. Bed net distribution activities should be accompanied by wide health education campaigns and followed up with tracking surveys to evaluate their effectiveness.

## Background

In Sudan, malaria is a major public health problem, topping the list of the main causes of morbidity, mortality and hospital attendance [1]. One of the key interventions to control malaria is vector control, including the use of insecticide treated nets (ITNs), the other key interventions being diagnosis and effective treatment of malaria cases as well as Indoor Residual Spraying (IRS) [2]. Studies have shown that bed nets do in fact reduce child mortality by about 20% and reduce the episodes of malaria by about 50% [3]. In addition, insecticide-treated nets also kill or keep away other insects, such as head lice, bedbugs and fleas [4] and have been useful in the prevention of other diseases such as leishmaniasis [5].

One of the main factors which affect the efficacy of ITNs is their retreatment every six to 12 months with insecticides. In response to the low retreatment rates of the bed nets, especially in Africa, Long-Lasting nets (LLINs) have been developed which require no further treatment throughout their expected life span of about three years or even more, making them more convenient and preferred over the conventional ones [2]. One main challenge is the price; not everybody can afford to buy LLINs. In Sudan, this has been solved by the free distribution of LLINs in 11 states as part of GFATM round 2. Studies in other countries have shown some problems in terms of utilization of insecticide-treated nets, especially during hot seasons [6].

The Olyset™ Net is a polyethylene net with 2% permethrin incorporated within the fibres. Over time, insecticide migrates to the surface of the yarn, replacing the one that has been removed by washing [7]. This is one of the LLINs recommended by the WHO and distributed in Sudan by the Malaria Control Programme. Some mosquito species, such as *Aedes aegypti *[8]*and Anopheles arabiensis *[9] were found to be resistant to permethrin in some studies. This raised concerns about the resistance of *Anopheles *mosquitoes to permethrin. There are also concerns about the effectiveness of LLINs a year or more after their distribution. A study in Tanzania [10] has concluded that Olyset™ bed nets were popular, durable and with a much longer insecticide persistence than ordinary polyester nets.

All these facts have made it apparent that a similar study needs to be conducted in Sudan to evaluate LLINs. This is why we attempted to conduct this research.

## Methods

### Study design

This is a two-step community-based study which examines the retention and efficacy of LLINs and factors that might influence them.

### Study area/setting

Kassala state lies 611 km east of Khartoum, the capital. With an average Annual Rainfall of 350–400 mm, the prevalence of malaria in Kassala state is 57/1000 population, carried predominantly by *An. arabiensis *mosquitoes. The malaria control programme in Kassala State Ministry of Health is working hard to fight this problem. They have started distributing LLINs since 2003. About 119,000 LLINs have been distributed since then, achieving coverage of about 5.7% [11]. This study was carried out in Kassala district (locality) which, as reported by the authorities, had coverage by LLINs of 90%.

### Sample size, Sampling type, and procedure

A sample of 210 households has been calculated using the 30 by 7 cluster sampling technique described by the WHO [12]. This is a two-stage cluster sampling. First, 30 clusters were selected from a total of 39 villages in which the bed nets were distributed more than one year ago. This was done with probability proportional to size (PPS). Then in each cluster, the first household was selected randomly from the list of houses. The other 6 houses within each cluster were then selected starting from the closest house to the first moving to the next closest and so on.

According to the protocol [13], four bed nets are enough to test for bioassay efficacy. We planned to collect 10 bed nets. These were to be collected from 10 households randomly selected from the households surveyed. It was agreed that if a bed net was not found in the house selected, a bed net should be taken from the following house. On four occasions, no bed nets were found in the selected house as well as the houses next to it; thus we were able to collect only six bed nets. This, however, should not have a major effect on the significance of the tests of efficacy since, as stated above, the required number of nets is four.

### Data collection

Data was collected by trained personnel using a pre-coded and pre-tested questionnaire. The questionnaire contained questions related to the retention of the bed nets, the utilization and perceptions related to the nets, as well as certain practices related to the nets, mainly retreating and washing.

Focus Group Discussions (FGD) were conducted in four clusters to complement and further explain the information obtained through the questionnaire. The clusters were selected giving consideration to the size of the area and the population, preference being given to areas of higher population density. In each cluster, two separate groups were selected: males and females. The groups ranged generally from about eight people to about twelve. Key issues and questions raised by results from the questionnaires were put forth for discussion. The questions focused on the distribution of the nets (the mechanism, price, number per household, and instructions to the recipients), the practices and utilization of the nets (including selling the nets, focus on children under five years of age and pregnant women), as well as pros and cons of the nets. The participants were encouraged to discuss freely while refraining from giving leading phrases to guide their response. All discussions have been audio recorded.

Bioassay efficacy was measured according to protocol of the National Malaria Control Programme, which was adapted from the WHO protocol. In this test, mosquitoes were exposed to the bed net material for three minutes. They were then placed inside plastic cups covered with netting and with sugar solution. They were then observed for Knock down (KD) after 60 minutes and mortality after 24 hours.

### Data management and analysis

Epi Info (Version 3.3.2) was used for data entry, data cleaning, and analysis. Quantitative data was summarized using proportions and means. Comparison of proportions between categorical variables was performed by a chi-squared test. Significance was predetermined at the 5% level.

### Ethical considerations

Institutional clearance was sought from the ministry of health before commencing the study. In the field, verbal informed consent was sought from all respondents. Every household from which a bed net is taken was given a new LLIN in exchange.

## Results

The informants were mainly males, 21–45 years old. More than half (57.6%) of the respondents have not received any formal education; in fact, none of the participants had any post-graduate studies. The income for the majority was below the national limit for extreme poverty of 300 Sudanese pounds per month per household.

### Retention

Olyset™ nets have been distributed to 142 households (70%) among those studied. Of those who received Olyset™ nets, 131 households (92.9%) had retained the nets until the time of the study (confirmed by observation) (Table 1).

**Table 1:** Retention, Utilization, and Efficacy of LLINs, Kassala State, Sudan, 2008

		**Frequency**	**Percentage**	**95% CI**	
**Retention **(n = 141)		131	92.9%	87.3%	96.5%
**Utilization**	**Children Under 5 **(n = 80)	44	55.0%	43.5%	66.2%
	**Pregnant Women **(n = 19)	8	42.1%	20.3%	66.5%
**Bioassay Efficacy**	**Knock Down after 60 minutes**		91.1%		
	**Mortality after 24 hours**		99.4%		

With regards to Olyset™ nets, 99 households (69.7%) have received one net, while 11 households (7.7%) have received three nets or more. In most areas, bed nets were distributed free of charge; however, people had to pay for the bed nets in some areas. Thirty (21.1%) of the households did not receive the nets for free and had to pay for them. The sum paid per bed net ranged from 3 to 5 Sudanese pounds, with 90% paying 3 pounds. This was considered to be the cost of the distribution by the local authorities.

The focus group discussions revealed that the distribution of bed nets was through many channels, varying according to the geographical areas. In general, the bed nets were not distributed by the malaria program in the ministry of health, but the local authorities and committees distributed the nets to the population living in each area. Most households received one bed net, a few received two, while fewer still received more than two bed nets. Some households have not received any bed nets at all. Most of the participants stated that they would like the number of bed nets per household to be increased.

In 25 (21.4%) households bed nets received were totally damaged (22 households) or sold (2 households) or missed (1 household). Forty-three nets (30.5%) had holes in them. None of the participants in the focus group discussions admitted that they, or others they knew, sold their bed nets.

There was a statistically significant relationship (p = 0.0171) between the level of education and the number of bed nets received, with those who have not received formal education receiving more nets than those who have. Those who have not received formal education were, however, more likely to have had lost their bed nets (not statistically significant). Bed net retention was significantly higher among those who received formal education (Fisher's exact test: p = 0.0104) (Table 2). The two households which have sold bed nets were those in which the informant had not received formal education.

**Table 2:** The Effect of Education Level and Income on the retention and utilization of LLINs, Kassala State, Sudan, 2008

		**Education**		
		
		**Received Formal Education**	**Did Not Receive Formal Education**	**P Value**
**Retention **(n = 141)		98.6% (68/69)	87.5% (63/72)	0.0104
**Utilization**	**Children Under 5 **(n = 80)	71.1% (32/45)	34.3% (12/35)	0.0179
	**Pregnant Women **(n = 19)	53.3% (8/15)	0% (0/4)	0.0415

		**Monthly Income Per Household**		
		
		**300 Pounds Or More**	**Less Than 300 Pounds**	**P Value**

**Retention **(n = 141)		100% (31/31)	90.9% (100/110)	0.0816
**Utilization**	**Children Under 5 **(n = 80)	70.6% (12/17)	50.8% (32/63)	0.7325
	**Pregnant Women **(n = 19)	50% (3/6)	38.5% (5/13)	0.7398

No statistically significant relationship was found between income and bed net retention.

### Utilization

Of the households interviewed, 196 (98.5%) thought that impregnated bed nets were useful, 163 (83.6%) of which giving the main reason for this being that the nets reduced the incidence of malaria. Discussions revealed that the majority also realized that the priority was for children under five years of age and pregnant women.

Eighty-two (58.2%) households reported sleeping under the bed nets every night. In 44 (55%) of the households with children under the age of five years, all children have slept under a bed net the previous night, while in 31 (38.8%) households none of the children under 5 years has slept under the bed net.

In eight (42.1%) of the households with pregnant women, these women have slept under bed nets, while in 10 households (52.6%), none of them has slept under a bed net the night before the interview.

The focus group discussions revealed that most of the participants did not know whether the bed nets were impregnated or not, and whether or not they needed to be reimpregnated. Twenty-seven households (19.4%) have reimpregnated their bed nets. Of these, 21 (77.8%) did this because they believed that the nets required reimpregnation. Ninety-one (65%) have washed their nets. Of these, 41 (46.6%) have washed their nets three times or more.

Participants in the FGD stated that bed nets were mainly used during and after the rainy season when the mosquitoes were abundant, but were not using them at the time of the study. The size of the bed nets were considered by some to be too large; they preferred smaller sizes; others thought that the large size was a main advantage, especially when compared with other types of nets. A few people thought that the mesh size was too big, but the majority did not think the size of the mesh was a problem. Only two women have stated that they were sensitive to the material in the bed nets.

Sixty-three (44.7%) households reported that at least one member of the household has suffered an attack of malaria (both clinical and/or lab-confirmed) during the past three months (Range: 1–6 bouts; Mean: 2.25). Seventeen (29.8%) households reported that those affected were sleeping under bed nets.

No statistical relationship was found between education level and the practice of reimpregnation of bed nets. Neither was a relationship found between education level and washing of bed nets. Of those who received education, 73.9% slept regularly under bed nets, compared to 43.1% among those who did not receive education (p < 0.01).

The percentage of households where all children under 5 slept under bed nets was significantly higher in households in which informants received education compared to those who did not (71.1% Vs 34.3%; p = 0.0179). Similar was the case for the percentage of pregnant women who slept under bed nets (53.3% Vs 0%; p = 0.0415). No significant difference was found concerning the perception of both groups about the usefulness of Olyset™ nets.

Also, no statistically significant relationship was found between income and practices of bed net reimpregnation or bed net washing. Across the different levels of income, no statistically significant difference was found for those who slept under bed nets or for percentages of children under 5 or pregnant women who slept under bed nets.

### Bioassay efficacy

The results of the bioassay efficacy tests revealed that knock down (KD) after 60 minutes ranged from 73.3% to 100% (Mean: 91.1%; SD: 10.037) while mortality after 24 hours in five bed nets was 100% and in one bed net was 96.66%. No significant relationship was found between the number of times the bed nets were washed and either the knock down or the mortality of the mosquitoes tested.

## Discussion

The fact that only 70% of the sample state that they have received LLINs contradicts the figures in the records. The retention among those who received the LLINs, found to be just below 93% was lower than that found in other similar studies [10]. This raises questions about the health education delivered to the population about the importance of having the bed nets as a few were damaged or sold.

According to the malaria directorate, the bed nets should be distributed to the households mainly according to the number of children under five years of age in the household. If a household has one or two children, they should receive one bed net; if more children are present, they should receive two. No household should have received more than two bed nets. However, in our sample, some houses have received three or more nets. After review of the data, this was found to be mostly unjustified, as most of those who received three or more bed nets did not have more than two children under five years of age and had no pregnant women in the house. On the other hand, some who had more than two children received one bed net. This is similar to problems witnessed in other countries concerning equity in bed net distribution [14].

Olyset™ nets cost more than the conventional ITNs [10]. However, with the support of the government and some organizations such as UNICEF, Olyset™ bed nets are provided free to the population. However, it is clear that some nets are still distributed for a cost. This might be one of the reasons why some households did not receive bed nets. It might also be the reason which tempted some households to sell the bed nets.

The fact that some households have reimpregnated the nets in the belief that they do in fact need to be reimpregnated raises once more questions about the messages delivered by those who distributed the nets. It also raises questions about the identity and experience of those who conduct and provide for the reimpregnation of the nets. These activities were conducted, according to the focus group discussions, by some NGOs.

Percentages for sleeping under bed nets in general and specifically for children under five years of age and for pregnant women are all quite low, lower than rates in nearby countries such as Eritrea [15]. This undermines the effectiveness of the bed nets as they are not being utilized regularly and are not being reserved and utilized by the main target groups.

All these factors might help explain the attacks of malaria within the past three months in 44.7% of the households. The fact that just under 30% of those affected were reported sleeping under the bed nets suggests problems with proper utilization. Some families mount the bed nets only late at night before they go to sleep, providing the mosquitoes with ample time to bite the inhabitants of the house [16].

The analyses point in general to a significant relationship between the education level of the informant and the practices related to LLINS. Those who have not received formal education actually received more bed nets. One possible reason for this might be that those who pursued their education were not as available as others who did not during the time of distribution of the nets. The facts that those who did not receive formal education were more likely to have lost their nets and that those who sold their nets belonged to this group raise other possible explanations. If bed nets are viewed just as a commodity to be obtained and then sold for financial gain, this could be a reason for asking for or buying more bed nets and then selling them. It was also observed that some houses misused the bed nets for purposes other than the one intended for them. All these facts suggest that those who received the nets did not fully appreciate the benefits and importance of the bed nets.

The effect of education is reflected clearly in the figures related to the utilization of the nets, with those receiving education sleeping more under the bed nets and letting more under 5s and pregnant women sleep under the bed nets.

The effect of the average monthly income on the different variables was not as significant as the relationship with education, except for those who reimpregnated their nets. Paradoxically, those with lower income reimpregnated their nets more than those with higher income.

The focus group discussions were quite useful in elucidating some of the confusing results from the questionnaires. Distribution of the bed nets by personnel who have not been trained by the malaria control program could explain why bed net distribution did not follow the same pattern in all areas, and why the coverage was not complete in all areas. In some areas some people received more bed nets than they were supposed to, while others did not receive any bed nets. It also explains why people had to pay to get the bed nets in some areas, and why the amount of money people had to pay varied from one area to another.

Along with the bed nets, the malaria control program handed the local authorities handouts containing information and instruction about using the bed nets. Since the local authorities did not receive training on properly delivering this information, many of the participants probably did not know some of the key information about the bed nets, e.g. whether or not they needed to be reimpregnated. Thus, the handouts might not have been as effective as was intended by the authorities. All these factors probably played a synergistic role which did not help ensure the retention and proper utilization of the nets.

However, the effect the bed nets had on repelling and killing the mosquitoes, eventually decreasing the incidence of malaria, was well observed by the population. Messages by the ministry of health about children under 5 and pregnant women being the priority groups to be targeted by bed nets were well received and clear to the participants. The benefits of the nets were very clear and most people did not think there were any disadvantages for the bed nets and they actually were asking for more information and more bed nets.

The results of the bioassay efficacy tests showed that the bed nets are still efficacious one-and-half years after distribution since mortality was more than 80% after 24 hours. This is similar to the results of the study in Tanzania [10]. Washing did not have a significant effect on knock down or mortality. This may be due to the fact that the bed nets did not get washed many times.

## Conclusion

The retention of bed nets was quite high but the coverage was lower than expected. The percentages of those sleeping under the bed nets, including the target group of children under five years of age and pregnant women were quite low. With mosquito knock down after 60 minutes at 91.1%, and mortality after 24 hours at 99.4%, Olyset™ bed nets have been shown to be fully efficacious.

## List of abbreviations

FGD: Focus Group Discussion; IRS: Indoor Residual Spraying; ITN: Insecticide treated bed net; KD: Knock Down; LLIN: Long-Lasting Insecticide-Treated Net; NGO: Non-Governmental Organization; RBM: Roll Back Malaria; WHO: World Health Organization

## Competing interests

The authors declare that they have no competing interests.

## Authors' contributions

SHH participated in the design of the study and the data collection, performed the statistical analysis, and helped draft the manuscript. EMM conceived of the study, and participated in its design and coordination and helped to draft the manuscript. SIO participated in the design of the study and the data collection. EME participated in the design and coordination of the study and helped to draft the manuscript. All authors have approved the final manuscript.
